# Adjuvant trastuzumab regimen for HER2-positive early-stage breast cancer: a systematic review and meta-analysis

**DOI:** 10.1080/17512433.2019.1637252

**Published:** 2019-07-09

**Authors:** Anne Julienne Genuino, Usa Chaikledkaew, Due Ong The, Thanyanan Reungwetwattana, Ammarin Thakkinstian

**Affiliations:** a Faculty of Pharmacy, Faculty of Medicine Ramathibodi Hospital, Faculty of Medicine Siriraj Hospital, Faculty of Public Health, Faculty of Social Sciences and Humanities, Institute for Population and Social Research, Faculty of Graduate Studies, Mahidol University Health Technology Assessment (MUHTA) Graduate Program, Bangkok, Thailand; b Social Administrative Excellence Research (SAPER) Unit, Department of Pharmacy, Faculty of Pharmacy, Mahidol University, Bangkok, Thailand; c Division of Medical Oncology, Department of Medicine, Faculty of Medicine Ramathibodi Hospital, Mahidol University, Bangkok, Thailand; d Section for Clinical Epidemiology and Biostatistics, Faculty of Medicine Ramathibodi Hospital, Mahidol University, Bangkok, Thailand

**Keywords:** Adjuvant chemotherapy, breast cancer, HER2-positive, meta-analysis, systematic review, trastuzumab

## Abstract

**Objective**: Breast cancer remains to be the globally leading female cancer. About 15% to 20% of breast cancers have human epidermal growth factor receptor 2 (HER2)-positive tumors – a more aggressive breast cancer subtype with shortened survival. In the light of new and updated trial data on trastuzumab therapy for HER2-positive early-stage breast cancer (EBC), we conducted a systematic review and meta-analysis to update the pooling of its relative treatment effects.

**Methods**: Systematic search was performed through Pubmed and Scopus to identify studies comparing survival outcomes and risks of heart toxicity effects of adjuvant trastuzumab with chemotherapy versus chemotherapy alone for HER2-positive EBC patients.

**Results**: Based on the eight included studies in the review, combining trastuzumab with chemotherapy continues to show lowered death and relapse risks by one-third. The decision to initiate trastuzumab, however, needs to be prudently deliberated as two to three times more cardiotoxicity risk was shown to be associated with its use.

**Conclusion**: Administering adjuvant trastuzumab in a weekly cycle concurrently with anthracycline-taxane chemotherapy regimen appears to be a preferable option to optimize its favorable effect in improving DFS and to prevent significantly higher risk for cardiotoxic effects.

## 1. Introduction

Breast cancer remains to be the leading cancer among women across the world with approximately 1.67 million cases and 521,907 deaths in 2012 as for the GLOBOCAN cancer incidence, mortality and prevalence report [1]. The World Health Organization reported that in 2008, almost 50% (691,300/1,383,500) of breast cancer cases and 58% (268,900/458,400) of breast cancer deaths occurred in less developed countries, contrary to the notion that breast cancer was a disease of developed countries [2]. They further reported that survival rates for breast cancer varied greatly worldwide, i.e., 80%, 60%, and 40% survival rates in high-income, middle-income and in low-income countries, respectively [3]. Limitations in detection and treatment facilities in less developed countries resulted in a large proportion of women with late diagnosis and thus treatment managements; thereby contributing to the poor prognosis in these countries [2].

Human epidermal growth factor receptor 2 (HER2) is a tyrosine kinase receptor that facilitates signaling pathways of cell growth, division, motility and repair [4]. Women with HER2-positive cells are more likely to have a more aggressive form of breast cancer, thus they tend to increase the risk of disease progression and decrease overall survival (OS) [4,5]. Hence, the determination of HER2 positivity through immunohistochemical (IHC) assay or in situ hybridization (ISH) assay became clinically important. The prevalence of HER2-positive breast cancer in Western population was approximately 15% to 20% of primary breast cancers [6], which was not much different compared to the estimated HER2 positivity rate in Asian population which was about 23.5% [7].

Currently, the 2018 American Society of Clinical Oncology (ASCO) [8], the 2017 US National Comprehensive Cancer Network (NCCN) Guideline on Breast Cancer [9], and the 2015 European Society for Medical Oncology (ESMO) Clinical Practice Guidelines for the Diagnosis, Treatment, and Follow-Up of Primary Breast Cancer [10] recommended the administration of trastuzumab (the first monoclonal antibody-based therapy developed to specifically target HER2) with chemotherapy for the management of HER2-positive early-stage breast cancer (EBC) in adjuvant settings. Trastuzumab may be administered by 3-week cycle (i.e., initial dose of 8 mg/kg, followed by 6 mg/kg every 3 weeks) or by weekly cycle (i.e., Initial dose 4 mg/kg, followed by 51 further weekly doses of 2 mg/kg), and sequentially or concurrently with standard chemotherapy of anthracycline-taxane regimens (e.g., doxorubicin and cyclophosphamide (AC) plus paclitaxel/docetaxel; or, fluorouracil, epirubicin and cyclophosphamide (FEC) plus docetaxel/paclitaxel), taxane regimen (e.g., docetaxel plus cyclophosphamide), or anthracycline regimen (e.g., AC). The treatment is recommended to start within two to six weeks after the surgery [9,10].

Trastuzumab is currently available as 150 mg (single-dose vial) and 440 mg (multi-dose vial) powder for concentrate solution for intravenous (IV) infusion; and for subcutaneous (SC) injection containing 600 mg/5mL. It works by binding to the juxtamembrane domain of HER2 thereby triggering the downregulation of HER2 expression. However, the treatment effect of adjuvant trastuzumab among EBC women with HER2-positive in improving the patient’s disease-free survival (DFS) and OS was controversial, as significant adverse cardiac effects (i.e., congestive heart failure, CHF and left ventricular ejection fraction, LVEF decline) were reported [11–13].

As for our knowledge, there were seven meta-analyses (N = 4–6 randomized controlled trials, RCTs) [14–20] and three systematic reviews only (N = 5–6 RCTs) [21–23]. Results of the seven meta-analyses [14–20] indicated that adjuvant trastuzumab with chemotherapy in treating HER2-positive EBC patients significantly improved the OS and DFS when compared to chemotherapy alone with a reduction in the risk of mortality ranging from about 30% to 34%, and a reduction in the risk of recurrence ranging from 34% to 50%. However, none of them comprehensively explored which prognostic factors (i.e., cycle regimen, type of administration, and the combined chemotherapy type) might affect both the relative treatment efficacy and safety. Results of this may aid in establishing a recommended optimum treatment regimen for adjuvant trastuzumab for HER2-positive EBC patients. In addition, one new RCT and two updated RCT results have been published since the last meta-analysis. We therefore conducted a systematic review and meta-analysis to update the pooling of the relative treatment efficacy and safety of adjuvant trastuzumab plus chemotherapy compared to chemotherapy alone in HER2-positive EBC patients. Subgroup analysis by prognostic factors and intervention characteristics was explored.

## 2. Methods

### 2.1. Search strategy

The review protocol was developed following the Preferred Reporting Items for Systematic reviews and Meta-Analyses (PRISMA) guidelines [24] and was registered at PROSPERO with registration number CRD42017072583. Electronic searching for relevant studies published from inception to 8 July 2017 was performed through two electronic databases (i.e. Medline via PubMed and Scopus), without language restrictions using search terms and strategies described in Table S1 (Supporting Information).

### 2.2. Selection of studies

Two review authors (AJG and DTO) independently screened all the titles and abstracts that were identified in the systematic searching. The full text of potentially eligible studies with relevant abstracts and titles were retrieved and independently evaluated for eligibility using the set inclusion and exclusion criteria. All excluded studies were recorded and properly accounted for reasons for exclusion. Any disagreement between the selection results of review authors was resolved by discussion and consensus with the supervisor (UC).

### 2.3. Inclusion and exclusion criteria

RCTs and non-RCTs were included if they met the following criteria: participating patients were HER2-positive EBC covering stage I, IIA, IIB, and IIIA; assessed the relative treatment effects of adjuvant trastuzumab plus any chemotherapy regimen (i.e., anthracycline-taxane regime, anthracycline-only regimen, taxane-only regimen) regardless of the cycle regimen type, timing and duration of administration; compared with any chemotherapy alone regimen; and, assessed any of following primary outcomes, i.e., overall survival (OS) defined as the time since randomization to death from any cause, disease-free survival (DFS) defined as the time since randomization to the date of recurrence of tumor; and/or secondary outcomes, i.e., congestive heart failure (CHF) defined as New York Heart Association as class III-IV, and left ventricular ejection fraction (LVEF) decline which was defined as absolute decline of at least 10% from baseline LVEF or a decline to less than 50% or 55%.

Studies were excluded if they evaluated: among populations which included/combined local and metastatic HER2-positive breast cancer patients; trastuzumab in neoadjuvant trastuzumab settings; trastuzumab with other anti-HER2 drugs; trastuzumab compared with different dosages or duration of the same interventions; and, if data are insufficient for pooling after contacting the authors.

### 2.4. Data extraction and management

We constructed and used data extraction forms which were composed of five data domains including study characteristics, patient characteristics (e.g., demographic data and relevant cancer data), interventions (e.g., dosing and cycle regimen type, duration of therapy, type of trastuzumab administration with chemotherapy), chemotherapy comparator, and outcomes data (e.g., type of outcomes, frequency data, and summary statistics). Data under these key domains were extracted and recorded from each included study. Any inconsistency in the extraction of data or in the assessment of studies was resolved by consensus with the supervisor (UC).

### 2.5. Risk of bias assessment

Included RCTs were assessed for Risk of Bias using Cochrane Handbook for Systematic Reviews of Interventions [25], composed of the following domains: random sequence generation, allocation sequence, blinding, incomplete outcome data and selective outcome reporting. Each domain was rated as low risk if relevant methods and procedures being assessed based on the criteria are adequate; high risk if procedures are inadequate; and unclear risk if unclear or not explicitly reported in the manuscript. Further, observational studies were assessed for quality using the Newcastle Ottawa Scale for Quality Assessment [26], which is composed of the following domains: selection of cohorts, comparability of cohorts and outcome assessment. Each domain includes item questions complemented with a scoring algorithm to rate each domain as good, fair or poor depending on the score that the study attained by meeting the set criteria for low risk of bias. The studies were then rated as having a low, moderate or high risk of bias if the percentage score was ≥75%, 50–74% and <50%, respectively. Two reviewers (AJG and DTO) independently assessed each study using the tools and pre-specified criteria. The supervisor (UC) was consulted for any disagreement in the ratings of the assessment.

### 2.6. Data synthesis

HR was used to assess relative treatment effects on OS and DFS, while RR was used to measure the safety outcomes CHF and LVEF decline. A meta-analysis of aggregated data from published studies was performed to pool the relative treatment effects using a random-effects model by DerSimonian and Laird method if heterogeneity was present (i.e., Cochran’s Q test <0.10 or I^2^ ≥ 25%); otherwise, a fixed-effects model by inverse variance method was used. Sources of heterogeneity were further explored by fitting the co-variables (i.e., tumor size, nodal, hormone-receptor status, type of intervention, trastuzumab duration, regimen cycle, administration type, length of follow-up period and the risk of bias) one-by-one in a meta-regression model. A decrease in I^2^ by at least 50% would suggest that such co-variable might be a source of heterogeneity. Sub-group analysis was then performed if data were available and sufficient.

A net clinical benefit was applied to assess risk (i.e., CHF and LVEF decline) and benefit (i.e., OS and DFS) simultaneously [27]. For this, risk difference (i.e., incremental (Δ)) was estimated for each study and pooled across studies using a random-effects model. An incremental risk and benefit ratio (IRBR) was then estimated. In addition, data for Δrisk and Δbenefit were further simulated using Monte Carlo with 1,000 replications assuming normal distributions for both Δrisk and Δbenefit. Finally, a cost-effective plane curve was constructed assigning Δrisk on Y-axis and Δbenefit on X-axis.

All the analyses were performed using STATA software version 14 and Microsoft excel version 2016. Two-sided P < 0.05 was considered statistically significant except for the subgroup analysis and heterogeneity test, in which P < 0.10 was used. Funnel plot and Egger’s test were used to detect potential publication bias. If any of these suggested asymmetry, a contour-enhanced funnel plot was constructed to further explore the likely attributable source of such asymmetry.

## 3. Results

### 3.1. Search results

**Figure 1.**
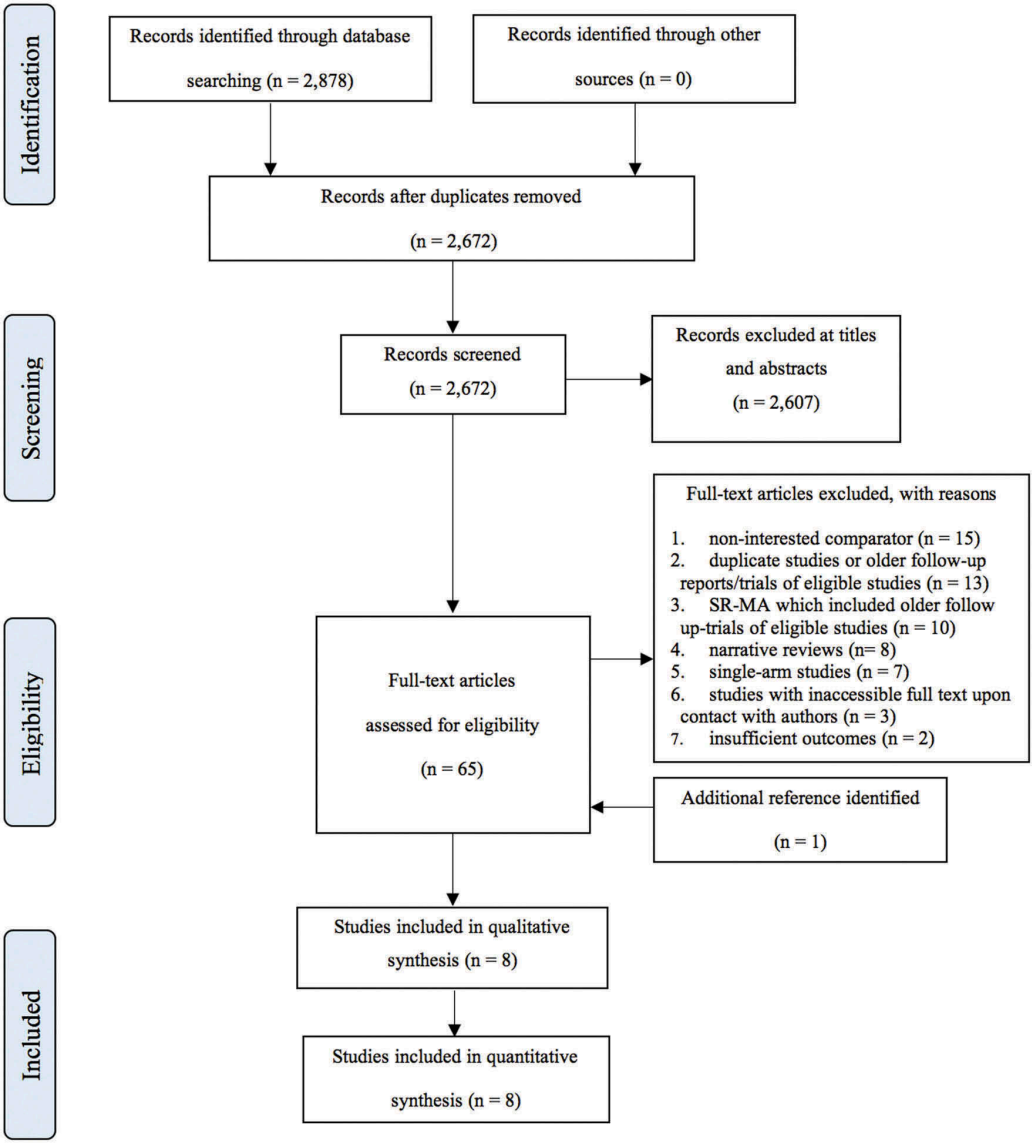
Study selection flow diagram.

Figure 1 illustrates the study flow of this systematic review. The combined electronic search from Medline and Scopus provided a total of 2,878 records. After removing the duplicates, 2,672 studies were remained for the screening of titles and abstracts. Of these, 2,602 studies were excluded leaving 65 studies plus one additional study from reference lists, for full text assessment. Of these, 58 studies did not meet the inclusion criteria, leaving eight studies included in the review [28–35].

### 3.2. Study characteristics

Study and patient characteristics of included studies are summarized in Table 1. All the included studies were RCTs. None of the detected non-RCTs met our criteria because their patients in the comparator group were mixed with chemotherapy and no treatment. Of these, three studies [32–34] were conducted in Europe, two studies [28,35] were international multicentre trials with collaborated countries from the different regions, and three studies [29–31] were from USA. The median follow-up period ranged from 3.9 to 11 years. The number of participating HER2-positive EBC women ranged from 232 to 4,046 with median age of 48 to 62 years. Majority of the included patients had a tumor size of more than 2 cm, while only three studies [28,32,33] provided information on the proportion of patients whose tumor size is less than 1 cm but with no report on their corresponding outcomes. The percentage of node-positive patients ranged from 52% to 100%, and hormone-receptor (HR)-positive patients were 40% to 73%. The percentage of patients who underwent mastectomy ranged from 36% to 67%.

**Table 1.** Study and patient characteristics.

		Study Characteristics			Patient Characteristics					
Author, Publication Year	Study Name	Study Design	Country	Median Follow up Period (years)	N	Median Age (yrs.)	Size of Tumor: % 2 cm or less	Nodal Status: % Positive Nodes	HR Status: % HR(+)	% Mastectomy
Cameron et al, 2017 [26]	HERA	RCT	39 countries from Europe, Canada, South Africa, Australia, New Zealand, Asia Pacific, Japan, Eastern Europe, Central and South America	11	3399	49	38.96 1cm: 0 %	57.30	50	57.83
Perez et al, 2014 [27]	N9831 +B31	RCT	USA	8.4	4046	-	39.53	93.03	54.75	38.98
Advani et al 2016 [29]	N9831	RCT	USA	9.2	1944	49	-	-	-	-
Romond et al 2012 [28]	B31	RCT	USA	7	2119	49	-	-	-	-
Joensuu et al, 2014 [33]	FinXX	RCT	41 countries from Europe, South Africa, Australia, South America, Asia Pacific	5.4	284	Int grp: 52.2 Control grp: 50.5	37.67	83.45	57.04	68.66
Slamon et al, 2011 [30]	BCIRG006	RCT	Finland	6.7	2147	49	39.80 1cm: 4.61%	71.38	54	60.7
Joensuu et al, 2009 [31]	FinHER	RCT	Finland	5.2	231	50.9	34.91 1cm: 6.90%	89	72.2	-
Spielmann et al, 2009 [32]	PACS-04	RCT	France and Belgium	3.9	528	48	43.55	100	40.34	36.36

N = sample size; HR = Hormone-Receptor; HR(+) = Hormone-Receptor Positive; RCT = Randomized controlled trial;

Information on intervention and comparator characteristics are summarized in Table S2 and S3 (Supporting Information). Four studies [29–33] administered trastuzumab in a 3-week cycle while three studies [28,34,35] implemented the weekly cycle. Courses of treatment were 12 months for seven studies [28–32,34,35] and 9 weeks for one study [33]. Trastuzumab was administered with chemotherapy concurrently in five studies [29–33] and sequentially in three studies [28,34,35]. Note that concurrent use of trastuzumab in our review refers to its concomitant use with chemotherapy regimen and not with the anthracycline drug component of the chemotherapy regimen as the latter is contraindicated due to irreversible cardiotoxicity. Hence, trastuzumab is used sequentially with anthracyclines drugs. For the type of chemotherapies, six studies [29–33,35] used anthracycline-taxane chemotherapy regimens, while the remaining two studies [28,34] used any/mixed type of chemotherapy regimens. None of the studies have used the same exact chemotherapy regimen combination. The comparators of all studies were the same chemotherapy regimen but without trastuzumab.

### 3.3. Risk of bias assessment of included studies

Risk of bias assessment was performed and results were described in Figure S4 (Supporting Information). Among eight studies, most studies, 5/8 [28–31,33] were rated as low risk for random sequence generation (N = 5 [28–31,33]), selective reporting (N = 7 [28–32,34,35]), and incomplete outcomes (N = 5 [28–31,33]). However, most studies (N = 6 [28–32,34]) were unclear whether they had applied allocation concealment, whereas none of the studies applied blinding.

### 3.4. Overall Survival (OS)

**Figure 2.**
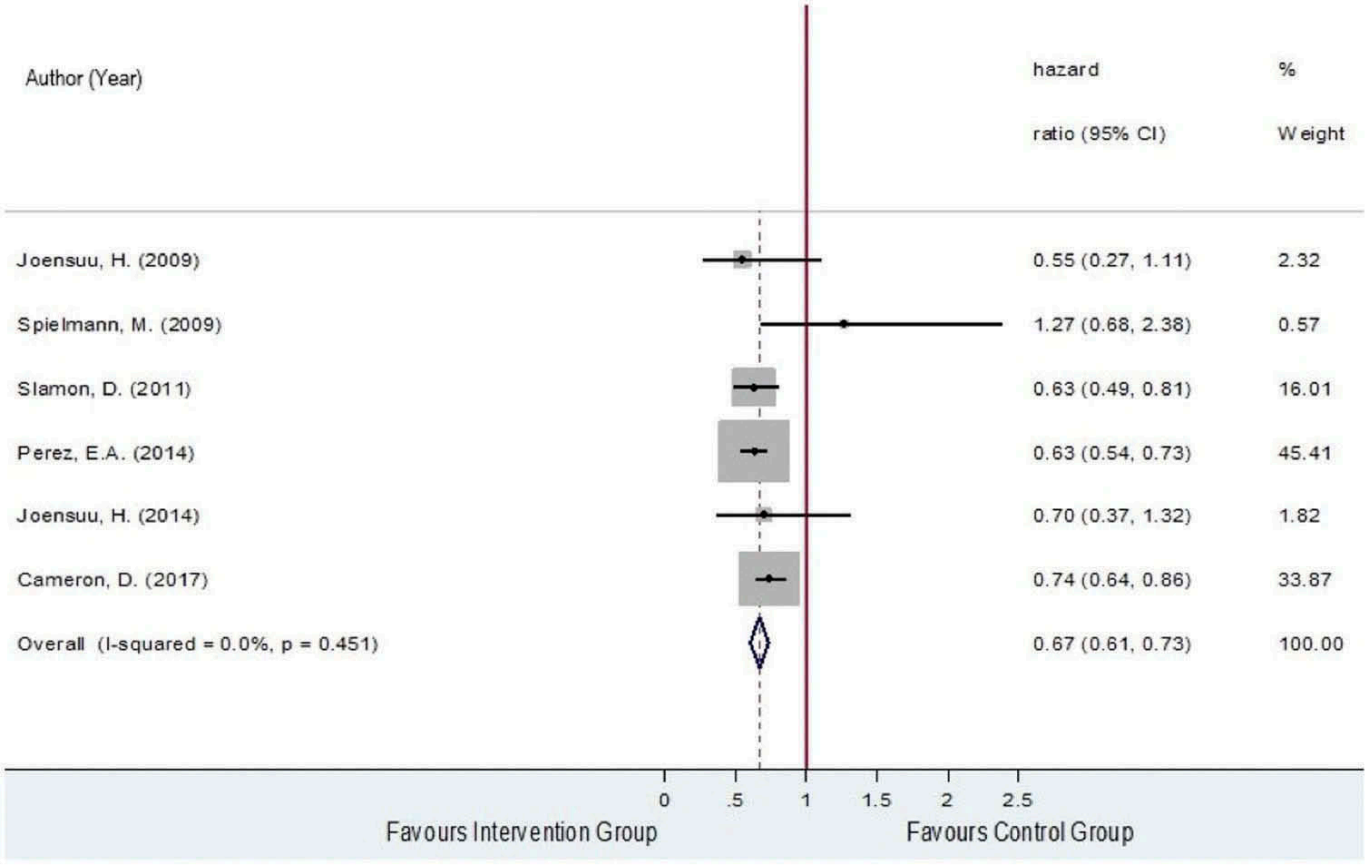
Forest plot for overall HR of OS.

The OS was obtained from six RCTs [28,29,32–35] which included a total of 5,355 and 5,280 of HER2-positive EBC women in the intervention and the control groups, respectively. Among these two groups, 736 and 1,023 patients died with pooled death rates and 95% confidence interval (CI) of 11.9% (8.5%, 15.5%) and 16.7% (11.4%, 22.0%). The reported hazard ratios (HRs) and 95% CIs of the individual studies are plotted in Figure 2 and showed that studies were generally in favor of the intervention except for Spielmann et al., 2009 [34]. The pooled HR was 0.67 (95% CI: 0.61, 0.73, P < 0.001) with a degree of heterogeneity of 0%, which could be interpreted that the risk of death was decreased by about 33% in the trastuzumab-chemotherapy group compared to the chemotherapy alone group.

### 3.5. Disease-Free Survival (DFS)

**Figure 3.**
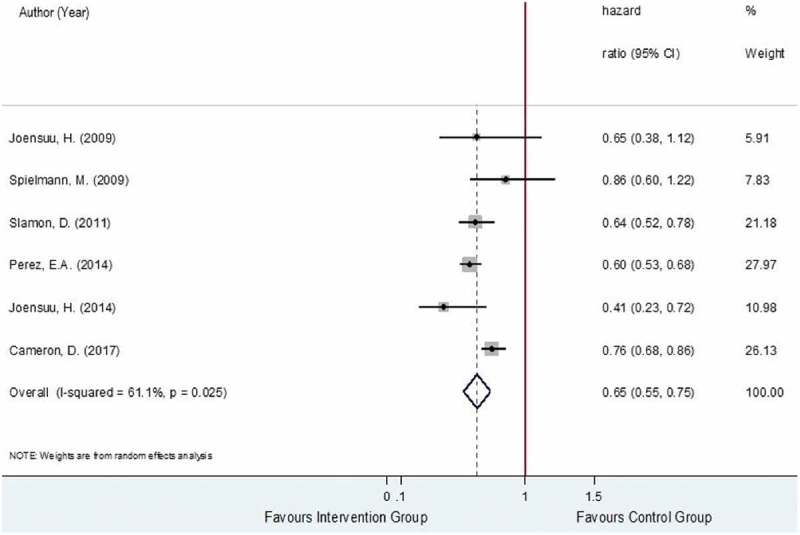
Forest plot for overall HR of DFS.

The DFS was estimated from six studies [28,29,32–35], analyzing a total of 10,635 HER2-positive EBC women. The total number of recurrences was, respectively, 1,271 out of 5,355 and 1,676 out of 5,280 in the trastuzumab-chemotherapy and chemotherapy alone groups with pooled recurrence rates (95% CI) of 21.6% (16.6%, 26.5%) and 29.4% (24.6%, 34.2%). The relative treatment effects of trastuzumab-chemotherapy regimen versus chemotherapy alone regimen on recurrence were moderately heterogeneous with the I^2^ of 61.1% as shown in Figure 3. The pooled HR was 0.65 (95% CI: 0.55, 0.75, P < 0.001), suggesting significantly lower risk of recurrence in the trastuzumab-chemotherapy group, about 35% when compared to the chemotherapy alone group.

Possible sources of heterogeneity in the pooling of HRs were then explored by performing subgroup analysis and among the pre-identified covariates, trastuzumab cycles (i.e., 3-week versus weekly cycles), intervention type (i.e., chemotherapy regimen combined with trastuzumab) and nodal status were found to be associated with the reduction in the degree of heterogeneity in some subgroups. For the subgroup analysis based on trastuzumab cycles, three RCTs [28,34,35] applied 3-week cycle and three RCTs [29,32,33] administered by weekly cycle. The results for the subgroup analysis based on cycle regimen apply for the subgroup analysis based on administration type as the trials belonging to the subgroups of the 3-week cycle and weekly cycle are similar for the subgroups of sequential and concurrent administration, respectively. The pooling within the subgroups of 3-week cycle versus weekly cycle yielded the I^2^ values of 73.9% and 0%, respectively, as shown in Figure S5 (Supporting Information). However, the pooled HRs for these corresponding groups were not much different with the pooled HRs of 0.68 (95% CI 0.44–0.91, P < 0.001) for the 3-week cycle and 0.61 (95% CI 0.55–0.68 P < 0.001) for the weekly cycle.

For the subgroup analysis based on intervention type, four RCTs [29,32,33,35] administered trastuzumab with anthracycline-taxane chemotherapy regimens while two RCTs [28,34] administered trastuzumab with any/mixed chemotherapy regimens (i.e., anthracycline-taxane chemotherapy, anthracycline-based chemotherapy, or taxane-based chemotherapy). The pooling of subgroups of trials which used trastuzumab with anthracycline-taxane chemotherapy regimen versus trastuzumab with any/mixed chemotherapy regimen type resulted in I^2^ values of 0% and 0%, respectively, as shown in Figure S6 (Supporting Information). The pooled subgroup HR for trastuzumab with any/mixed chemotherapy regimen type was significantly higher (HR 0.77; 95% CI: 0.68, 0.85, P < 0.001) compared to the pooled subgroup HR for trastuzumab with anthracycline-taxane chemotherapy regimen (HR 0.60; 95% CI: 0.54, 0.66, P < 0.001). This indicates that higher reduction in the risk of recurrence was observed in the intervention arm of trials which have administered trastuzumab with anthracycline-taxane chemotherapy regimens (i.e., 40%), as compared to the reduction in the risk of recurrence from the intervention arm of the trials which have administered trastuzumab with any/mixed chemotherapy regimen type (i.e., 23%).

For the subgroup analysis based on nodal status, five RCTs [29,32–35] involved 60% or more node-positive patients in their study while only one trial by Cameron et al., 2017 [28] involved less than 60% node-positive patients. The pooling of subgroup of trials involving 60% or more node-positive patients decreased the I^2^ to 25.5%, as shown in Figure S7 (Supporting Information). The pooled subgroup HR for trials which involved 60% or more node-positive patients was lower (HR 0.61; 95% CI: 0.53, 0.70, P < 0.001) compared to the HR of one trial with less than 60% node-positive patients (HR 0.76; 95% CI: 0.67, 0.85, P < 0.001) suggesting higher reduction in the risk of recurrence observed in the administration of trastuzumab among trials where more node-positive patients have participated (i.e., 39%), as compared to the reduction in the risk of recurrence of the trial with less participating node-positive patients (i.e., 24%).

The funnel plots for OS and DFS are provided in S8 and S9 (Supporting Information). The funnel plots and Egger’s tests for OS and DFS showed symmetry, both with non-statistically significant p values for Egger’s test, suggesting no evidence of publication bias.

### 3.6. Congestive Heart Failure (CHF)

**Figure 4.**
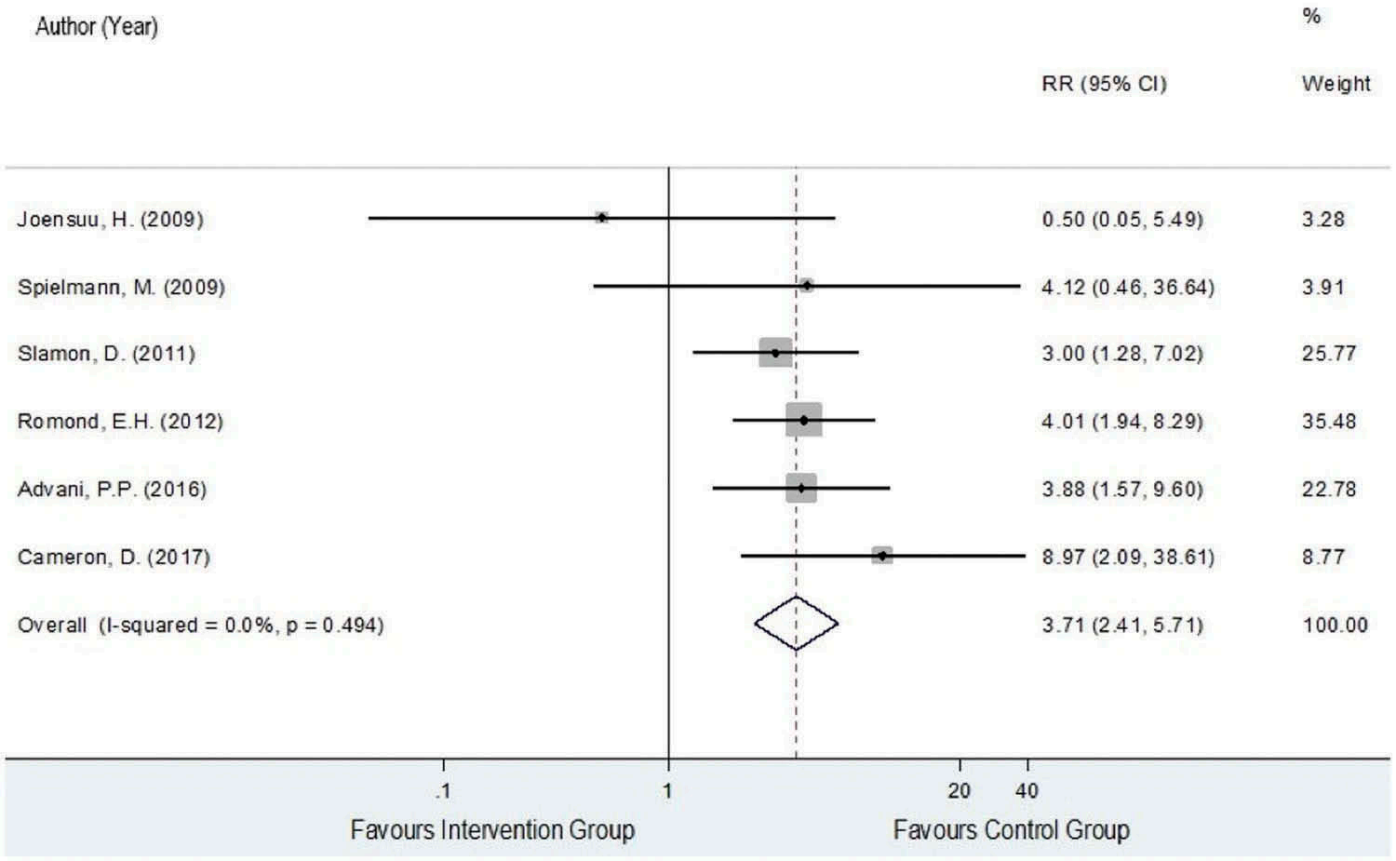
Forest plot for overall RR of CHF.

The data on CHF were obtained from six RCTs [28,30–34] analyzing a total of 4,779 and 4,879 HER2-positive EBC women in the trastuzumab-chemotherapy and the chemotherapy alone groups, respectively. A total of 100 and 79 patients of these corresponding groups developed CHF with pooled rates (95% CI) across studies of 2.0% (1.1%, 2.9%) and 0.6% (0.2%, 0.9%). The reported risk ratios (RRs) and 95% CIs of individual studies and showed generally higher CHF events in the intervention group compared to the control group except for Joensuu et al., 2009 [33], with a degree of heterogeneity of 0%. The pooled RR for CHF was 3.71 (95% CI: 2.41, 5.71, P < 0.001) as shown in Figure 4, which could be interpreted that the risk of CHF in the trastuzumab-chemotherapy group increased by 3.71 times more compared to the chemotherapy alone group.

### 3.7. Left Ventricular Ejection Fraction (LVEF) decline

**Figure 5.**
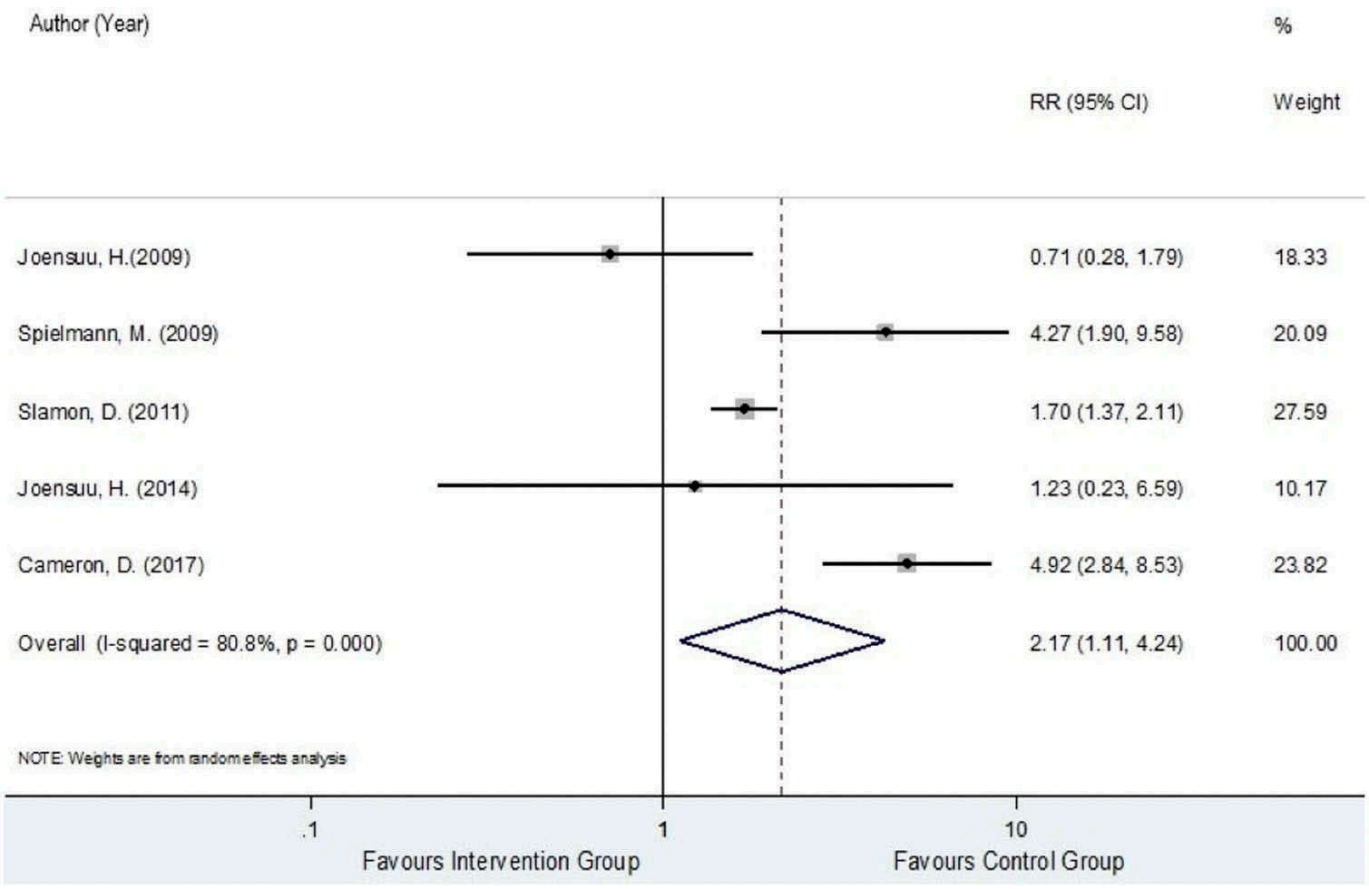
Forest plot for overall RR of LVEF Decline.

Data for LVEF decline were extracted from five trials [28,32–35] analyzing a total 3,327 and 3,262 HER2-positive EBC women in the trastuzumab-chemotherapy and the chemotherapy alone groups, respectively. Among these two groups, 308 and 148 developed LVEF decline with pooled rates across studies of 8.4% (2.5%, 14.2%) and 4.7% (0.7%, 8.8%). The reported RRs and 95% CIs of individual studies similarly showed that LVEF decline events were generally higher in the intervention group compared to the control group except for Joensuu et al., 2009 [33]. The relative treatment effects of trastuzumab-chemotherapy regimen versus chemotherapy alone regimen on the risk of LVEF decline were substantially heterogeneous with the I^2^ of 80.8%. The pooled RR was 2.17 (95% CI: 1.11, 4.24, P < 0.001), as presented in Figure 5, suggesting a significantly higher risk of LVEF decline in the trastuzumab-chemotherapy group by 2.17 times more when compared to chemotherapy alone group.

Possible sources of heterogeneity in the pooling of RR for LVEF decline were then explored and among the pre-identified covariates, trastuzumab cycle regimen, administration type, and intervention type (i.e., chemotherapy regimen combined with trastuzumab) were found to be associated. For the subgroup analysis of LVEF decline based on trastuzumab cycle regimen, three RCTs [28,34,35] administered by 3-week cycle and two RCTs [32,33] using the weekly cycle. Similarly, the results for the subgroup analysis based on cycle regimen apply for the subgroup analysis based on administration type. The pooling within subgroups of 3-week cycle versus weekly cycle resulted in the I^2^ values of 15.5% and 69.2%, respectively, as presented in Figure S10 (Supporting Information). The pooled RR for the 3-week cycle was significantly higher (RR 4.16; 95% CI: 2.50, 6.92, P < 0.001) compared to the pooled RR for the weekly cycle (RR 1.24; 95% CI: 0.54, 2.83, P = 0.614).

For the subgroup analysis of LVEF decline based on intervention type, three RCTs [28,32–35] administered trastuzumab with anthracycline-taxane chemotherapy regimen while two RCTs [28,34] administered trastuzumab with any/mixed chemotherapy regimen type yielding the I^2^ values of 40% and 0%, respectively, as shown in Figure S11 (Supporting Information). The pooled RRs of these two corresponding subgroups were 4.70 (95% CI: 2.98, 7.41, P < 0.001) and 1.32 (95% CI 0.74–2.36, P = 0.350), respectively.

### 3.8. Risk and benefit assessment

Net clinical benefits were assessed to simultaneously compare risks (i.e., CHF and LVEF decline) and benefits (i.e., OS and DFS). Incremental risks and benefits were estimated for individual included studies and then pooled across studies using a random-effects model, see Figure S12 (Supporting Information). IRBRs were further estimated, i.e., 0.1895, 0.3005, 0.5135, and 0.8144 for ΔCHF/ΔDFS, ΔCHF/ΔOS, ΔLVEF decline/ΔDFS, and ΔLVEF decline/ΔDFS, respectively (see Table S13 [Supporting Information]); the lower IRBR reflects the better benefit over the risk. Comparing with CHF, the IRBRs were about 1/5 and 1/3, i.e., DFS and OS would be one-fifth and one-third or more than the development of CHF. For LVEF decline, the IRBRs were a half and four-fifth, i.e., DFS and OS were about 50% and 20% or lower than the development of LVEF decline. Monte Carlo with 1000 replications was applied to simulate data Δrisks and Δbenefits, cost‐effective plane curves were constructed, see Figure S14 a-d (Supplemental Information). If the threshold is set at 0.5, receiving trastuzumab-chemotherapy should gain benefits from DFS and OS than the risk of CHF, but not for LVEF decline.

## 4. Discussion

We conducted a systematic review and meta-analysis of eight RCTs which demonstrated that adding adjuvant trastuzumab to chemotherapy compared to chemotherapy alone in HER2-positive EBC patients can reduce the risk of mortality and recurrence of 33% and 35%, respectively. However, adjuvant trastuzumab might result in three and two times increased risk of CHF and LVEF decline compared to using the standard chemotherapy regimens alone. Net clinical benefit was assessed considering risk and benefit simultaneously which showed that DFS and OS were about one-fifth and one-third or more than the development of CHF, whereas DFS and OS were about a half and one-fifth or lower than the development of LVEF decline.

We updated the previous meta-analyses by including new and latest RCTs with the longest follow up period report to date [28–31,35]. Our results were consistent with the results of previous meta-analyses [14–20] which showed risk reduction for death and recurrence by about 30% to 34% and 36% to 50%, respectively. Likewise, our pooled results showed increasing risk of CHF and LVEF decline by about 3.71 and 2.17 times, respectively, which corresponded with previous meta-analyses results of 2.45 to 7.60 times for CHF and 1.88 to 2.45 times for LVEF decline. We, however, have added more evidence that receiving adjuvant trastuzumab therapy might gain benefits of DFS and OS about one-fifth and one-third or more over adverse drug reactions of CHF compared with receiving chemotherapy alone. While our study excluded neoadjuvant settings and focused on trastuzumab adjuvant use as it is the first and more widely used indication especially among developing countries, many patients in developed countries are currently treated with neoadjuvant trastuzumab therapy and dual blockade trastuzumab with pertuzumab.

Hence, judicious decision on the trade-offs between these beneficial and unfavorable treatment effects remain to be an important consideration prior to deciding the initiation of the therapy. Given this result and the fact that trials included in this analysis have all excluded patients with pre-existing cardiovascular disease or low LVEF level (i.e., less than 50%), it is mandatory to assess cardiovascular functions prior to initiation and continuously monitoring during the trastuzumab-chemotherapy therapy.

Moreover, this analysis explored on several factors which may possibly affect both the treatment efficacy and safety outcomes. Among the assessment of factors related to the characteristics of the patients, study and the intervention regimens, the results have shown the association of treatment effect (i.e., DFS and LVEF decline) with the type of intervention regimen particularly the chemotherapy regimen type combined with trastuzumab, the cycle regimen type of trastuzumab, and the type of trastuzumab administration.

The subgroup analyses showed improved DFS or significantly higher reduction in the risk of recurrence when adjuvant trastuzumab was administered in a weekly cycle concurrently with anthracycline-taxane chemotherapy regimens. As regards the type of trastuzumab administration, the results of this updated analysis was consistent with the previous results by Yin et al., 2011 [18] showing better DFS improvement for concurrent administration (OR 0.65, 95% CI 0.49–0.85) compared to sequential administration (OR 0.74, 95% CI 0.65–0.83).

In terms of cardiotoxic outcomes, the subgroup analyses also showed that the risk of LVEF decline appeared to be significantly higher when adjuvant trastuzumab was administered in a 3-week cycle sequentially with any/mixed type of chemotherapy regimens but the risks were dramatically declined with the weekly cycle regimen, concurrent administration and trastuzumab combined with anthracycline-taxane chemotherapy. However, these latter poolings might be limited by the small number of included studies and thus lack of statistical power.

In this light, cardiotoxic outcomes associated with trastuzumab use remains to be an important decision point by clinicians and patients, regardless of the cycle regimen, administration type and intervention regimen. On the basis of these comparative subgroup results weighing both the efficacy and safety outcomes, adjuvant trastuzumab administered in a weekly cycle concurrently with anthracycline-taxane chemotherapy regimens appears to be a preferable option in terms of optimizing its beneficial treatment effect as shown by its higher risk reduction for relapse, and in terms of averting higher risk for detrimental outcomes as shown by its lesser magnitude of risk of LVEF decline.

Further research is recommended to explore and determine which particular chemotherapy regimen type combined with trastuzumab provides the best improvement in survival outcomes and least risk of cardiotoxic effects towards identifying the best treatment regimen for this therapy. Assessment of optimal treatment regimen based on other relevant patient-related outcomes (e.g., quality of life) as well as cost-effectiveness and the role of biosimilars which can lower the cost of therapy to improve its accessibility especially among resource-constrained settings are likewise strongly recommended. Moreover, it is speculative that in the future the related research in this field will evolve to provide clear evidence on the role of adjuvant trastuzumab among HER2-positive patients with small node-negative tumors which could lead to the improvement of clinical practice for these patients.

## 5. Conclusion

In the light of the most current and longest trial evidence available to date, combining adjuvant trastuzumab with chemotherapy is able to gain the benefits of OS and DFS over the risk of CHF but not for LVEF decline when compared to chemotherapy alone in HER2-positive EBC women. The currently available evidence under the subgroup analyses showed that administering adjuvant trastuzumab in a weekly cycle concurrently with anthracycline-taxane chemotherapy regimen is able to lower cardiogenetic toxicity than 3-week cycle given not much differences in the benefit of OS and DFS.

## Article Highlights

- HER2-positive breast cancer poses significant clinical burden among breast cancer patients with its more aggressive disease phenotype resulting in poorer prognosis.
- Trastuzumab has been recommended for the treatment of HER2-positive breast cancer but itis associated with cardiotoxic effects.
- An updated systematic review and meta-analysis along with exploration on the impact of prognostic factors and intervention characteristics, and net clinical benefit was performed in aid of establishing a recommended optimum treatment regimen for adjuvant trastuzumab for HER2-positive patients.
- Current evidences available to date indicated that combining adjuvant trastuzumab with chemotherapy might significantly improve the OS and DFS of HER2-positive EBC women thereby decreasing the risk for mortality and relapse by one-third, but also increases the risk for cardiotoxic effects by two to three times more.
- Combining adjuvant trastuzumab with chemotherapy might gain benefits of DFS and OS about one-fifth and one-third or more than CHF.
- Administering adjuvant trastuzumab in a weekly cycle concurrently with anthracycline-taxane chemotherapy regimen appears to be a preferable option to optimize its favorable effect in improving DFS and to prevent significantly higher risk for cardiotoxic effects.

## References

[1] World Health Organization International Agency for Research on Cancer. GLOBOCAN 2012: estimated Cancer incidence, mortality and prevalence worldwide 2012 [cited 2017 323]; Available from: http://globocan.iarc.fr/Pages/fact_sheets_cancer.aspx.

[2] World Health Organization Breast cancer: prevention and control. World Health Organization; 2017 [cited 2017 323]; Available from: https://www.who.int/cancer/detection/breastcancer/en/

[3] Coleman MP, Quaresma M, Berrino F, et al Cancer survival in five continents: a worldwide population-based study (CONCORD). Lancet Oncol. 2008;9(8):730–756. 18639491 10.1016/S1470-2045(08)70179-7

[4] Burstein H. The distinctive nature of HER2-positive breast cancers. N Engl J Med. 2005 10 20;353(16):1652–1654. 16236735 10.1056/NEJMp058197

[5] Gajria D, Chandarlapaty S HER2-amplified breast cancer: mechanisms of trastuzumab resistance and novel targeted therapies. Expert Rev Anticancer Ther. 2011;11(2):263–275. 21342044 10.1586/era.10.226 PMC3092522

[6] Wolff AC, Hammond ME, Hicks DG, et al Recommendations for human epidermal growth factor receptor 2 testing in breast cancer: American society of clinical oncology/College of American pathologists clinical practice guideline update. J Clin Oncol. 2013;31(31):3997–4013. 24101045 10.1200/JCO.2013.50.9984

[7] Pathmanathan N, Geng JS, Li W, et al Human epidermal growth factor receptor 2 status of breast cancer patients in Asia: results from a large, multicountry study. Asia Pac J Clin Oncol. 2016;12:369–379. 27334915 10.1111/ajco.12514

[8] Giordano SH, Temin S, Chandarlapaty S, et al Systemic therapy for patients with advanced human epidermal growth factor receptor 2–positive breast cancer: ASCO clinical practice guideline update. J Clin Oncol. 2018 9 10;36(26):2736–2740. 29939838 10.1200/JCO.2018.79.2697

[9] National Comprehensive Cancer Network NCCN Guidelines version 2.2017. Breast Cancer; 2017; Pennsylvania, PA: National Comprehensive Cancer Network, Plymouth Meeting.

[10] Senkus E, Kyriakides S, Ohno S, et al Primary breast cancer: ESMO clinical practice guidelines for diagnosis, treatment and follow-up. Ann Oncol. 2015;26(Suppl 5):v8–V30. 26314782 10.1093/annonc/mdv298

[11] Romond EH, Perez EA, Bryant J, et al Trastuzumab plus adjuvant chemotherapy for operable HER2-positive breast cancer. N Engl J Med. 2005;353(16):1673–1684. 16236738 10.1056/NEJMoa052122

[12] Slamon D, Eiermann W, Robert N, et al, Phase III randomized trial comparing doxorubicin and cyclophosphamide followed by docetaxel (AC→T) with doxorubicin and cyclophosphamide followed by docetaxel and trastuzumab (AC→TH) with Docetaxel, Carboplatin and Trastuzumab (TCH) in Her2neu positive early breast cancer patients: BCIRG 006 study. San Antonio Breast Cancer Symposium. 353(16):1652–1654, 2009.

[13] Smith I, Procter M, Gelber RD, et al 2-year follow-up of trastuzumab after adjuvant chemotherapy in HER2-positive breast cancer: a randomised controlled trial. Lancet. 2007;369(9555):29–36. 17208639 10.1016/S0140-6736(07)60028-2

[14] Bria E, Cuppone F, Fornier M, et al Cardiotoxicity and incidence of brain metastases after adjuvant trastuzumab for early breast cancer: the dark side of the moon? A meta-analysis of the randomized trials. Breast Cancer Res Treat. 2008;109(2):231–239. 17638068 10.1007/s10549-007-9663-z

[15] Viani GA, Alfonso SL, Stefano EJ, et al Adjuvant trastuzumab in the treatment of her-2-positive early breast cancer: a meta-analysis of published randomized trials. BMC Cancer. 2007;7:153. 17686164 10.1186/1471-2407-7-153 PMC1959236

[16] Dahabreh IJ, Linardou H, Siannis F, et al Trastuzumab in the adjuvant treatment of early-stage breast cancer: a systematic review and meta-analysis of randomized controlled trials. Oncologist. 2008;13(6):620–630. 18586917 10.1634/theoncologist.2008-0001

[17] Ward S, Pilgrim H, Hind D Trastuzumab for the treatment of primary breast cancer in HER2-positive women: a single technology appraisal. Health Technol Assess. 2009;13(Suppl. 1.). 10.3310/hta13suppl1/01 19567207

[18] Yin W, Jiang Y, Shen Z, et al Trastuzumab in the adjuvant treatment of HER2-positive early breast cancer patients: a meta-analysis of published randomized controlled trials. PLoS ONE. 2011;6(6):e21030. 21695277 10.1371/journal.pone.0021030 PMC3111470

[19] O’Sullivan CC, Bradburry I, Campbell C, et al Efficacy of adjuvant trastuzumab for patients with human epidermal growth factor receptor 2–positive early breast cancer and tumors ≤ 2 cm: a meta-analysis of the randomized trastuzumab trials. J Clin Oncol. 2015 20;33(24):2600–2608. 26101239 10.1200/JCO.2015.60.8620 PMC4534523

[20] Long HD, Lin YE, Zhang JJ, et al Risk of congestive heart failure in early breast cancer patients undergoing adjuvant treatmentwith trastuzumab: a meta-analysis. Oncologist. 2016;21(5):547–554. 27026675 10.1634/theoncologist.2015-0424 PMC4861364

[21] Gonzalez-Angulo AM, Hortobagyi GN, Esteva FJ Adjuvant therapy with trastuzumab for HER-2/neu-positive breast cancer. Oncologist. 2006;11(8):857–867. 16951389 10.1634/theoncologist.11-8-857

[22] Madarnas Y, Trudeau M, Franek JA, et al Adjuvant/neoadjuvant trastuzumab therapy in women with HER-2/neu overexpressing breast cancer: A systematic review. Cancer Treat Rev. 2008;34(6):539–557. 18502589 10.1016/j.ctrv.2008.03.013

[23] Mates M, Fletcher GG, Freedman OC, et al Systemic targeted therapy for her2-positive early female breast cancer: a systematic review of the evidence for the 2014 cancer care Ontario systemic therapy guideline. Curr Oncol. 2015;22(Suppl1):S114–S22. 25848335 10.3747/co.22.2322 PMC4381787

[24] Moher D, Liberati A, Tetzlaff J, et al Preferred reporting items for systematic reviews and meta-analyses: the PRISMA statement. PLoS Med. 2009;6(7). 10.1371/journal.pmed.1000097 PMC2707599 19621072

[25] Higgins JP, Altman DG, Gøtzsche PC, et al The Cochrane Collaboration’s tool for assessing risk of bias in randomised trials. BMJ. 2011;343:d5928. 22008217 10.1136/bmj.d5928 PMC3196245

[26] Wells GA, Shea B, O’Connell D, et al The Newcastle-Ottawa Scale (NOS) for assessing the quality of nonrandomised studies in meta-analyses. 2014 [cited 2017 71]; Available from: http://www.ohri.ca/programs/clinical_epidemiology/oxford.asp.

[27] Towse A Net clinical benefit: the art and science of jointly estimating benefits and risks of medical treatment. Value Health. 2010 6;13(Suppl 1):S30–S32. 20618793 10.1111/j.1524-4733.2010.00753.x

[28] Cameron D, Piccart-Gebhart MJ, Gelber RD, et al 11 years’ follow-up of trastuzumab after adjuvant chemotherapy in HER2-positive early breast cancer: final analysis of the HERceptin Adjuvant (HERA) trial. Lancet. 2017;369(9555):29–36. 10.1016/S0140-6736(16)32616-2 PMC5465633 28215665

[29] Perez EA, Romond EH, Suman VJ, et al Trastuzumab plus adjuvant chemotherapy for human epidermal growth factor receptor 2–positive breast cancer: planned joint analysis of overall survival from NSABP B-31 and NCCTG N9831. J Clin Oncol. 2014;23(31):7811–7819. 10.1200/JCO.2014.55.5730 PMC4226805 25332249

[30] Romond EH, Jeong JH, Rastogi P, et al Seven-year follow-up assessment of cardiac function in NSABP B-31, a randomized trial comparing doxorubicin and cyclophosphamide followed by paclitaxel (ACP) with ACP plus trastuzumab as adjuvant therapy for patients with node-positive, human epidermal growth factor receptor 2–positive breast cancer. J Clin Oncol. 2012;30(31):3792–3799. 22987084 10.1200/JCO.2011.40.0010 PMC3478574

[31] Advani PP, Ballman KV, Dockter TJ, et al Long term cardiac safety analysis of NCCTG N9831 (Alliance) adjuvant trastuzumab trial. J Clin Oncol. 2016;23(31):7811–7819. 10.1200/JCO.2015.61.8413 PMC4980566 26392097

[32] Slamon D, Eiermann W, Robert N, et al Adjuvant trastuzumab in HER2-positive breast cancer. N Engl J Med. 2011;365(14):1273–1283. 21991949 10.1056/NEJMoa0910383 PMC3268553

[33] Joensuu H, Bono P, Kataja V, et al Fluorouacil, epirubicin and cyclophosphamide with either docetaxel or vinorelbine, with or without trastuzumab, as adjuvant treatments of breast cancer: final results of the FinHer trial. J Clin Oncol. 2009;27(34):5685–5692. 19884557 10.1200/JCO.2008.21.4577

[34] Spielmann M, Roche H, Delozier T, et al Trastuzumab for patients with axillary-node-positive breast cancer: results of the FNCLCC-PACS 04 trial. J Clin Oncol. 2009;27(36):6129–6134. 19917839 10.1200/JCO.2009.23.0946

[35] Joensuu H, Kellokumpu-Lehtinen P, Huovinen R, et al Outcome of patients with HER2-positive breast cancer treated with or without adjuvant trastuzumab in the finland capecitabine trial (FinXX). Acta Oncol. 2014;53(2):186–194. 23957715 10.3109/0284186X.2013.820840 PMC3894716

