# Supplementary material for: Adjuvant trastuzumab regimen for HER2-positive early-stage breast cancer: a systematic review and meta-analysis
Source: Expert Rev Clin Pharmacol. 2019 Jul 9;12(8):815–24. doi: 10.1080/17512433.2019.1637252 (PMC6816496; doi:10.1080/17512433.2019.1637252)
Supplement: Supplemental Material [file IERJ_A_1637252_SM9122.docx]

**Table S1. Search strategy framework and ineligibility coding**

| **P: HER2+ EARLY BREAST CA** | **AND**  **I: ADJUVANTTRASTUZUMAB (combined with Chemotherapy** | **C: Chemotherapy alone** | **O: Efficacy**  **Safety** |
| --- | --- | --- | --- |
| HER2 **OR** ERBB2 | Trastuzumab **OR** Herceptin  **AND** |  |  |
| **AND**  Breast Cancer **OR** Breast Neoplasm **OR** Breast Carcinoma | Adjuvant **OR** Post-operative Treatment |  |  |

Ineligible studies were accounted and coded using the following ineligibility coding:

|  | **0** | Duplicates |
| --- | --- | --- |
| **Study design** | **1** | Non-interested study design |
| **P** | **2** | Non interested population |
| **I** | **31** | Non interested intervention |
| **C** | **32** | No comparator/single arm studies |
|  | **33** | Non interested comparator |
| **O** | **41** | Non-interested outcomes |
|  | **42** | Insufficient outcome reporting |
| **Others** | **51** | Duplicated or older follow up trial reports of eligible studies |
|  | **52** | Editorials/ Commentary/ Literature Review/ Case series/ Case reports/ Narrative reviews/ Letter to Editor/ Proceedings/ protocol/ Position Paper/ Peer Review Summary/ Expert Consensus |
|  | **53** | Studies with inaccessible fulltext manuscript upon contact with author |
|  | **54** | Systematic Review and Meta-Analysis, Systematic Review, or Meta-Analysis including older follow up reports of eligible studies |

**Table S2. Intervention and comparator characteristics of included studies**

| Study Name | INTERVENTION | | | | COMPARATOR |
| --- | --- | --- | --- | --- | --- |
|  | Trastuzumab regimen | Duration of Therapy (months) | Type of Trastuzumab Administration | Type Chemotherapy combined with Trastuzumab | Type Chemotherapy comparator |
| **HERA** | Initial dose 8 mg/kg, followed 6 mg/kg every 3 weeks | 12 | Sequential | Mixed:  Anthracycline-based or Anthracycline-Taxane CT or Antimetabolites and Alkylating agents CT | Mixed:  Anthracycline-based or Anthracycline-Taxane CT or Antimetabolites and Alkylating agents CT |
| **N9831**  **+B31** | *Initial dose 4 mg/kg, followed by 51 further weekly doses of 2 mg/kg* | 12 | Concurrent | Anthracycline-Taxane CT *(Doxorubicin-based)* | Anthracycline-Taxane CT *(Doxorubicin-based)* |
| **N9831** | *Initial dose 4 mg/kg, followed by 51 further weekly doses of 2 mg/kg* | 12 | Concurrent | Anthracycline-Taxane CT *(Doxorubicin-based)* | Anthracycline-Taxane CT *(Doxorubicin-based)* |
| **B31** | *Initial dose 4 mg/kg, followed by 51 further weekly doses of 2 mg/kg* | 12 | Concurrent | Anthracycline-Taxane CT *(Doxorubicin-based)* | Anthracycline-Taxane CT *(Doxorubicin-based)* |
| **FinXX** | Initial dose 8 mg/kg, followed 6 mg/kg every 3 weeks  *Initial dose 4 mg/kg, followed 2 mg/kg weekly, nine cycles* | 12 months = 74.4%  2.25 months = 25.6% | *Sequential = 74.4%*  *Concurrent = 25.6%* | Anthracycline-Taxane CT  *(Epirubicin-based)* | Anthracycline-Taxane CT  *(Epirubicin-based)* |
| **BCIRG006** | *Initial dose 4 mg/kg, followed by 2 mg/kg per week during CT then 6mg/kg q3weeks* | 12 | Concurrent | Anthracycline-Taxane CT *(Doxorubicin-based)* | Anthracycline-Taxane CT *(Doxorubicin-based)* |
| **FinHER** | *Initial dose 4 mg/kg, followed 2 mg/kg weekly, nine cycles* | 2.25 | Concurrent | Anthracycline-Taxane CT *(Epirubicin-based)* | Anthracycline-Taxane CT *(Epirubicin-based)* |
| **PACS-04** | Initial dose 8 mg/kg, followed 6 mg/kg every 3 weeks | 12 | Sequential | Anthracycline-based or Anthracycline-Taxane CT *(Epirubicin-based)* | Anthracycline-based or Anthracycline-Taxane CT *(Epirubicin-based)* |

**CT – chemotherapy*

**Table S3.**  **Therapy regimen of intervention and control arms**

| **Study Name**  **Author Year** | **Intervention Group** | **Control Group** |
| --- | --- | --- |
| **HERA**  **Cameron et al, 2017** | **ANTHRACYCLINE-based regimen:**   - Doxorubicin/ epirubicin 🡪 cyclophosphamide 🡪 **TRASTUZUMAB** - Doxorubicin/ epirubicin 🡪 cyclophosphamide-fluorouracil 🡪 **TRASTUZUMAB** - Doxorubicin/ epirubicin 🡪 cyclophosphamide-methotrexate-fluorouracil 🡪 **TRASTUZUMAB** - Doxorubicin/ epirubicin -cyclophosphamide 🡪 cyclophosphamide-methotrexate-fluorouracil 🡪 **TRASTUZUMAB**   **ANTHRACYCLINE-TAXANE regimen:**   - doxorubicin-cyclophosphamide🡪 paclitaxel 🡪 **TRASTUZUMAB** - doxorubicin-cyclophosphamide🡪 docetaxel 🡪 **TRASTUZUMAB** - epirubicin-cyclophosphamide🡪 paclitaxel 🡪 **TRASTUZUMAB** - epirubicin-cyclophosphamide🡪 docetaxel 🡪 **TRASTUZUMAB**   **ANTIMETABOLITES and ALKYLATING AGENTS- regimen:**   - Cyclophosphamide-methotrexate-fluorouracil 🡪 **TRASTUZUMAB** | **ANTHRACYCLINE-based regimen:**   - Doxorubicin/ epirubicin 🡪 cyclophosphamide - Doxorubicin/ epirubicin 🡪 cyclophosphamide-fluorouracil Doxorubicin/ epirubicin 🡪 cyclophosphamide-methotrexate-fluorouracil - Doxorubicin/ epirubicin -cyclophosphamide 🡪 cyclophosphamide-methotrexate-fluorouracil   **ANTHRACYCLINE-TAXANE regimen:**   - doxorubicin-cyclophosphamide🡪 paclitaxel - doxorubicin-cyclophosphamide🡪 docetaxel - epirubicin-cyclophosphamide🡪 paclitaxel - epirubicin-cyclophosphamide🡪 docetaxel   **ANTIMETABOLITES and ALKYLATING AGENTS- regimen:**   - Cyclophosphamide-methotrexate-fluorouracil |
| **N9831+B31**  **Perez et al, 2014** | **doxorubicin-cyclophosphamide**^3-week cycle^ x 4 cycles 🡪 **paclitaxel** once a week (for 12weeks) or once every 3 weeks (for 4 cycles) concurrently with **TRAZTUZUMAB** once per week followed by **TRAZTUZUMAB** alone to complete one year of therapy | **doxorubicin-cyclophosphamide**^3-week cycle^ x 4 cycles 🡪 **paclitaxel** once a week (for 12weeks) or once every 3 weeks (for 4 cycles) |
| **N9831**  **Advani et al, 2016** | **doxorubicin-cyclophosphamide**^3-week cycle^ x 4 cycles 🡪 **paclitaxel** once a week (for 12weeks) or once every 3 weeks (for 4 cycles) concurrently with **TRAZTUZUMAB** once per week followed by **TRAZTUZUMAB** alone to complet  e one year of therapy | **doxorubicin-cyclophosphamide**^3-week cycle^ x 4 cycles 🡪 **paclitaxel** once a week (for 12weeks) or once every 3 weeks (for 4 cycles) |
| **B31**  **Romond et al, 2012** | **doxorubicin-cyclophosphamide**^3-week cycle^ x 4 cycles 🡪 **paclitaxel** once a week (for 12weeks) or once every 3 weeks (for 4 cycles) concurrently with **TRAZTUZUMAB** once per week followed by **TRAZTUZUMAB** alone to complete one year of therapy | **doxorubicin-cyclophosphamide**^3-week cycle^ x 4 cycles 🡪 **paclitaxel** once a week (for 12weeks) or once every 3 weeks (for 4 cycles) |
| **FinXX**  **Joensuu et al, 2014** | SEQUENTIAL:  **docetaxel** ^3-week-cycle^  x 3 cycles 🡪 **FEC** ^3-week cycle^ x 3 cycles 🡪 **TRASTUZUMAB** every 3 weeks for 12 months  OR  **docetaxel-capecitabine** ^3-week-cycle^  x 3 cycles 🡪 **cyclophosphamide- epirubicin-capecitabine** ^3-week-cycle^  x 3 cycles 🡪 **TRASTUZUMAB** every 3 weeks for 12 months    CONCURRENT:  **docetaxel** ^3-week-cycle^  x 3 cycles + **TRASTUZUMAB** for 9 weeks 🡪 FEC^3-week cycle^ x 3 cycles  OR  **docetaxel-capecitabine** ^3-week-cycle^  x 3 cycles + **TRASTUZUMAB** for 9 weeks 🡪 **cyclophosphamide-** **epirubicin-capecitabine** ^3-week-cycle^  x 3 cycles | **docetaxel** ^3-week-cycle^  x 3 cycles 🡪 **FEC**^3-week cycle^ x 3 cycles    OR    **docetaxel-capecitabine** ^3-week-cycle^  x 3 cycles 🡪 **cyclophosphamide- epirubicin-capecitabine** ^3-week-cycle^  x 3 cycles    **capecitabine on D1 and D15 of the cycle* |
| **BCIRG006**  **Slamon et al, 2011** | **doxorubicin-cyclophosphamide**^3-week cycle^ x 4 cycles 🡪 **docetaxel** every 3 weeks for 4 doses – **TRAZTUZUMAB** (once per week x 12 weeks) 🡪 **TRAZTUZUMAB** (every 3 weeks to complete 1 year) | **doxorubicin-cyclophosphamide**^3-week cycle^ x 4 cycles 🡪 **docetaxel** every 3 weeks for 4 doses |
| **FinHER**  **Joensuu et al, 2009** | **docetaxel**^3-week-cycle^  x 3 cycles + **TRASTUZUMAB** weekly for 9 weeks 🡪 **FEC** ^3-week cycle^ x 3 cycles  OR  **vinorelbine** weekly for 9 cycles + **TRASTUZUMAB** weekly for 9 weeks 🡪 **FEC** ^3-week cycle^ x 3 cycles | **docetaxel**^3-week-cycle^  x 3 cycles 🡪 **FEC** ^3-week cycle^ x 3 cycles  OR  **vinorelbine** weekly for 9 cycles 🡪 **FEC** ^3-week cycle^ x 3 cycles |
| **PACS-04**  **Spielmann et al, 2009** | **FEC** ^3-week cycle^ x 6 cycles 🡪 **TRASTUZUMAB** q3weeks x 18 cycles    OR    **E-Docetaxel** ^3-week cycle^ x 6 cycles 🡪 **TRASTUZUMAB**q3weeks x 18 cycles | **FEC** ^3-week cycle^ x 6 cycles    OR    **E-Docetaxel** ^3-week cycle^ x 6 cycles |

******FEC - fluorouracil, epirubicin, cyclophosphamide; E - epirubicin*

**Figure S4. Risk of bias summary for included studies**

**
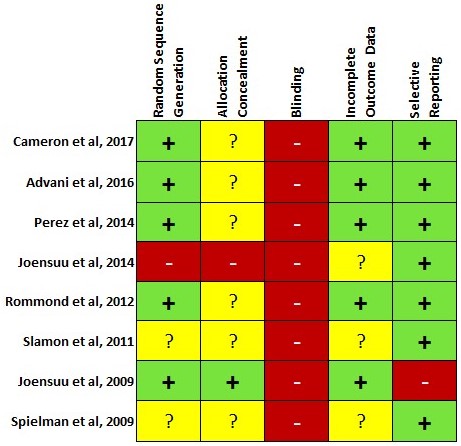
**


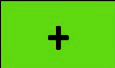


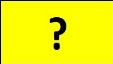
Low risk of bias


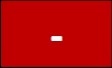
Unknown risk of bias

High risk of bias

**Figure S5. Subgroup analysis for Disease-Free Survival stratified by trastuzumab regimen cycle**

**
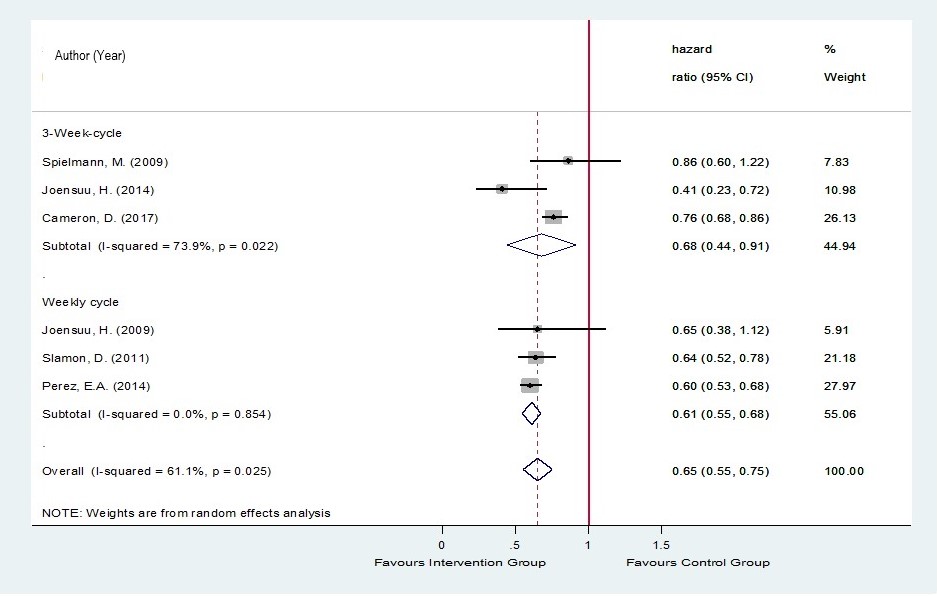
**

**Figure S6. Subgroup analysis for Disease-Free Survival stratified by intervention type**

**
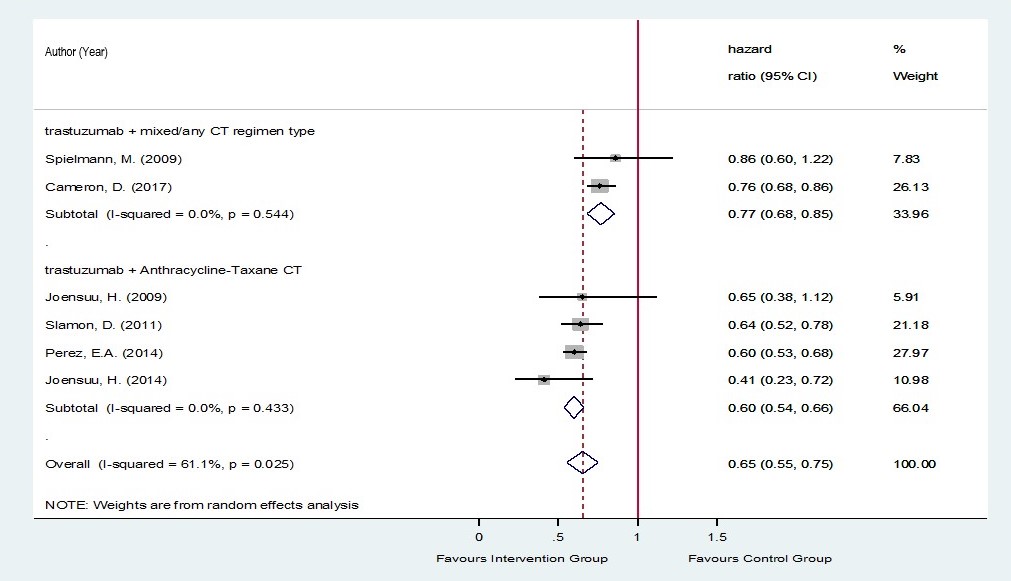
**

**CT – chemotherapy*

**Figure S7. Subgroup analysis of Disease-Free Survival stratified by nodal status**

***
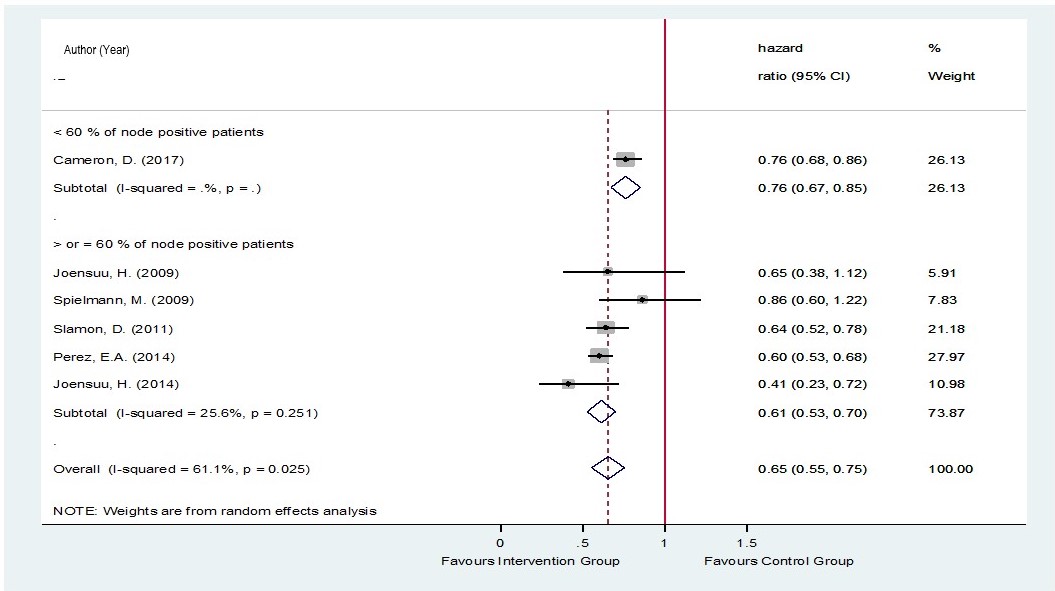
***

**Figure S8. Funnel plot for Overall Survival**

**
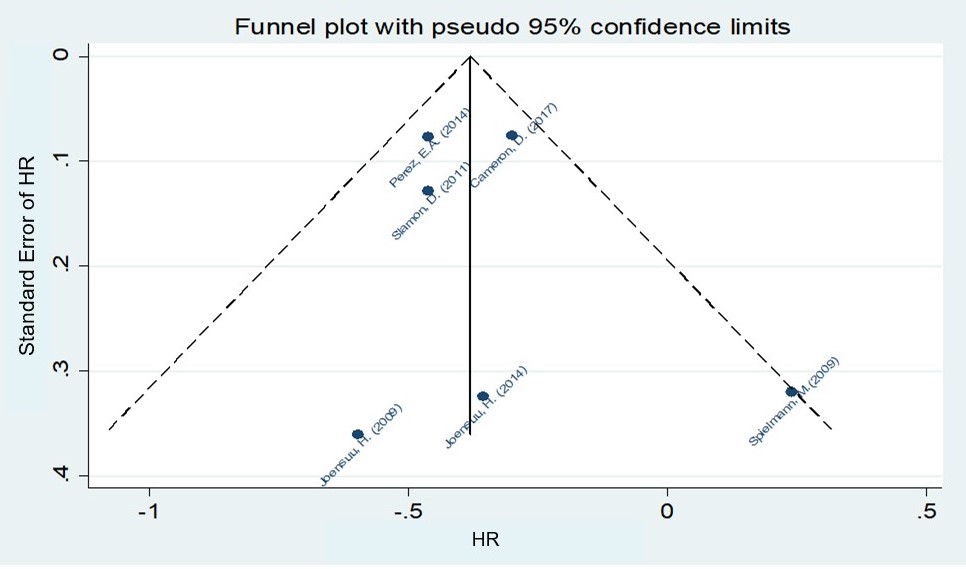
**

**Figure S9. Funnel plot for Disease-Free Survival**

**
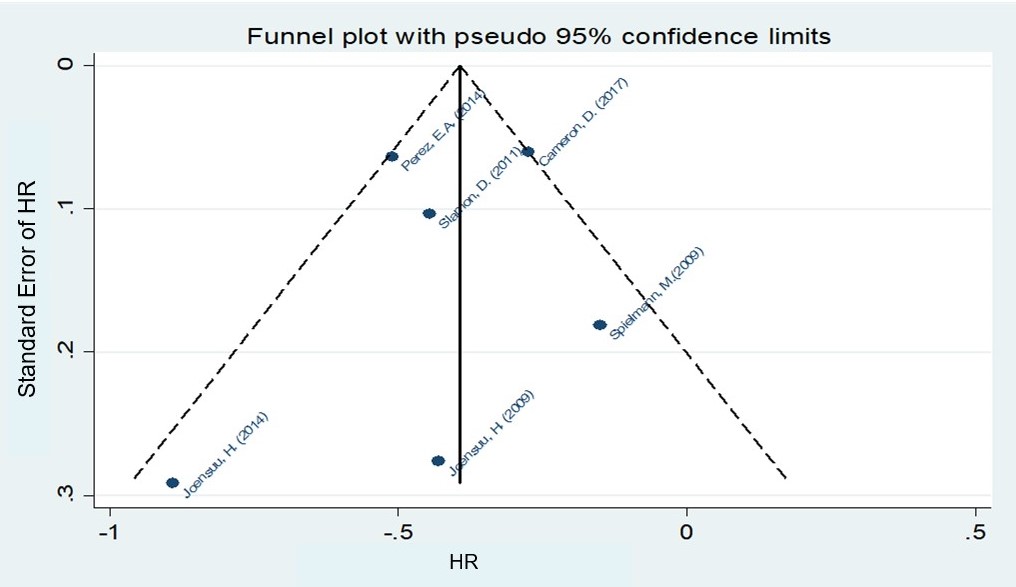
**

**Figure S10. Subgroup analysis on LVEF decline stratified by trastuzumab cycle regimen**

**
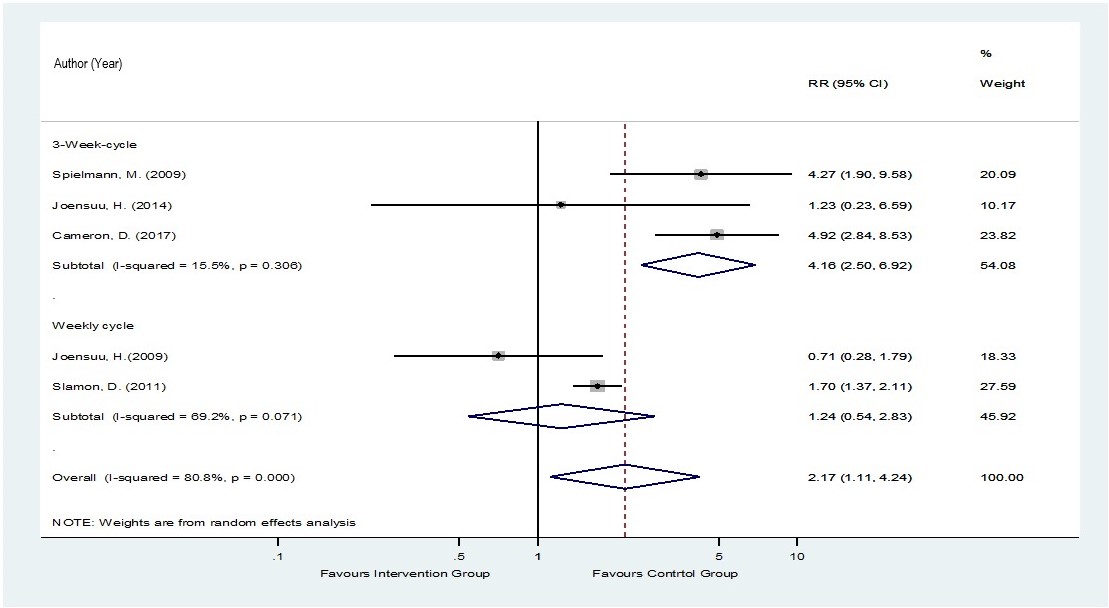
**

**Figure S11. Subgroup analysis on LVEF decline stratified by intervention type**

**
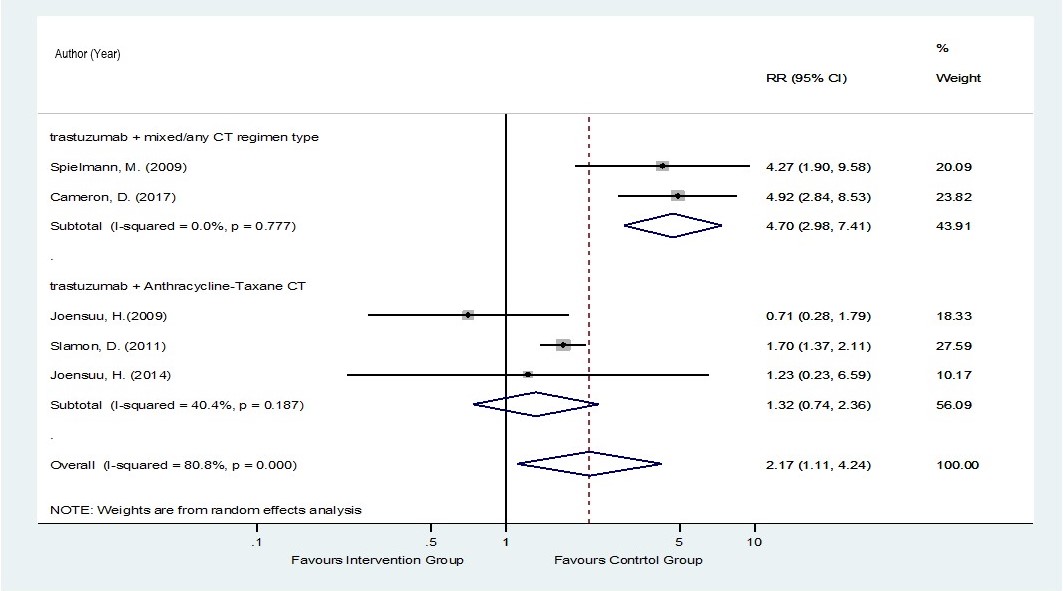
**

**CT – chemotherapy*

**Figure S12. Forrest plots of risk differences of OS, DFS, CHF, and LVEF decline between trastuzumab-chemotherapy and chemotherapy alone**

**Table S13. Estimations of incremental risks and benefits ratios between trastuzumab-chemotherapy and chemotherapy alone**

|  | TRZ+Chem | | | Chem alone | | | Incremental | Δrisk/Δbenefit | Δrisk/Δbenefit |
| --- | --- | --- | --- | --- | --- | --- | --- | --- | --- |
|  | event | n | Pooled rate | event | n | Pooled rate | Δ | DFS | OS |
| Benefit | | | | | | | | | |
| Death | 4619 | 5,355 | 0.8802 | 4257 | 5,280 | 0.8328 | 0.0483 |  |  |
| Recurrence | 4084 | 5,355 | 0.7844 | 3604 | 5,280 | 0.7064 | 0.0766 |  |  |
| Risk | | | | | | | | | |
| CHF | 100 | 4,779 | 0.0203 | 27 | 4,879 | 0.0056 | 0.0145 | 0.1895 | 0.3005 |
| LVEF  decline | 308 | 3,327 | 0.0837 | 148 | 3,262 | 0.0474 | 0.0393 | 0.5135 | 0.8144 |

**Figure S14. Cost-Effectiveness Plane Curves**

1. **IRBR (ΔCHF / ΔOS)**

1. **IRBR (ΔCHF / ΔDFS)**

1. **IRBR (ΔLVEF decline/ΔOS)**

1. **IRBR (ΔLVEF decline/ΔDFS)**
